# Profiling Osteoporosis via Integrated Multi-Omics Technologies

**DOI:** 10.3390/cells15050472

**Published:** 2026-03-05

**Authors:** Adriano Braile, Adriano Bani, Seyedeh Fatemeh Hosseininasab, Nicola del Regno, Nicola Orabona, Antonio Bove, Mariantonia Braile

**Affiliations:** 1 Unit of Orthopaedics and Traumatology, Ospedale del Mare, 80147 Naples, Italyorabona.nicola@aslnapoli1centro.it (N.O.);; 2 Department of Medical and Surgical Specialties and Dentistry, University of Campania “Luigi Vanvitelli”, 81100 Naples, Italy; 3 Department of Clinical Sciences and Translational Medicine, University of Tor Vergata, 00133 Rome, Italy; 4 UOSD Laboratorio Territoriale SS Annunziata, ASL Napoli 1, 80145 Naples, Italy; 5 Department of Molecular Medicine and Medical Biotechnologies, University of Naples Federico, 80145 Naples, Italy

**Keywords:** osteoporosis, multi-omics, transcriptomics, proteomics, metabolomics, epigenomics, bone–muscle axis, biomarkers, precision medicine

## Abstract

Background: Osteoporosis is a complex disorder involving bone loss and muscle degeneration. Multi-omics technologies provide novel insights into its molecular mechanisms and may support biomarker discovery, patient stratification, and therapeutic development. Objective: This scoping review aimed to synthesize current evidence on the application of multi-omics approaches in osteoporosis, focusing on molecular insights, methodological diversity, and translational potential. Methods: A literature search of PubMed, Embase, and Scopus retrieved 433 records using the keywords “osteoporosis,” “osteosarcopenia,” and “omics.” After removing duplicates and screening titles, abstracts, and full texts, 30 studies met the inclusion criteria. Data on study populations, biological samples, multi-omics techniques, and integration methods were extracted. Results: Studies employed transcriptomics, proteomics, metabolomics, lipidomics, epigenomics, and metagenomics, often combined in multi-omics analyses with computational modeling. Key pathways included osteoclast differentiation, immune regulation, ferroptosis, and microbiome–metabolite interactions. Multi-omics integration enabled the identification of molecular subtypes, candidate biomarkers, and potential therapeutic targets. Limitations included small or single-center cohorts, heterogeneous designs, and limited validation, restricting generalizability and clinical translation. Conclusions: Multi-omics approaches offer a powerful framework to uncover the molecular mechanisms underlying bone and muscle degeneration and to guide precision diagnostics and interventions. Future studies should prioritize large, multicenter, longitudinal designs integrating multi-omics data with clinical and functional validation to facilitate clinical application.

## 1. Introduction

Osteoporosis is a common condition affecting a substantial proportion of older adults worldwide. According to the World Health Organization (WHO), it is diagnosed when the bone mineral density (BMD) T-score is ≤−2.5 standard deviations, or based on alternative fracture-risk criteria [1,2,3]. The condition is associated with an elevated risk of fragility fractures, pain, disability, and mortality [3,4]. The term osteosarcopenia has been introduced to highlight the co-occurrence of osteoporosis (or low bone mass) and sarcopenia [3,5]. Sarcopenia is characterized by muscle atrophy, reduced strength, and functional decline [6]. Although historically regarded as distinct disorders, accumulating evidence demonstrates that bone and muscle are biologically interconnected: mechanically (muscle loading stimulates bone formation), through paracrine and endocrine signaling (myokines and osteokines), and by regulatory mechanisms such as stem-cell lineage allocation. Together, these processes contribute to the coupling of bone and muscle health [7].

Prevalence estimates for osteosarcopenia vary widely (~5% to 37%), depending on definitions and populations, but the condition consistently correlates with adverse clinical outcomes, including increased fracture risk, higher fall frequency, greater institutionalization, prolonged hospitalization, and elevated mortality [8,9]. Because diagnostic definitions and criteria are not standardized, early detection and intervention remain suboptimal. Moreover, most clinical settings address bone and muscle separately rather than as an integrated bone–muscle unit [10]. Established diagnostic tools for osteoporosis include dual-energy X-ray absorptiometry (DXA) for BMD assessment, fracture-risk estimators such as the Fracture Risk Assessment Tool (FRAX), and biochemical markers of bone turnover [11]. Sarcopenia assessment commonly relies on measurements of muscle mass (DXA, CT, MRI, or ultrasound), grip strength, and gait speed [6]. For osteosarcopenia, however, standardized diagnostic algorithms are lacking, and only a limited number of validated biomarkers or molecular diagnostic tools have been implemented in routine clinical practice [12].

Multi-omics technologies offer major advantages for elucidating the mechanisms governing bone and muscle health [13,14]. High-throughput molecular profiling enables deep interrogation of biological systems, including the identification of genetic variants, transcriptional signatures, epigenetic modifications, and proteomic or metabolomic patterns associated with skeletal or muscular decline [13,14]. A key strength of multi-omics approaches is their ability to provide a systems-level perspective, capturing the molecular networks linking bone and muscle with the immune system, metabolism, and the microbiome [13,14]. This integrative view enhances our understanding of both physiological regulation and pathological processes.

Multi-omics research also expands opportunities for personalized medicine [13]. By characterizing individual molecular profiles, it becomes possible to differentiate, for example, patients whose bone loss is driven by inflammatory pathways from those affected by stem-cell senescence [15]. Such molecular endotyping may inform more targeted preventive and therapeutic strategies. Furthermore, multi-omics analyses can detect molecular perturbations that arise before measurable phenotypic changes—such as reductions in BMD or muscle mass—indicating that multi-omics-derived biomarkers could facilitate earlier diagnosis and intervention.

By mapping critical nodes within molecular networks, multi-omics approaches can also uncover novel therapeutic targets—not only for bone or muscle individually but also for the complex bone–muscle crosstalk that underlies osteoporosis [16].

A growing body of research demonstrates the power of multi-omics in musculoskeletal science. For instance, integrated glycomics–genomics analyses have identified the transporter gene SLC10A7 as essential for proper bone mineralization through post-Golgi protein transport and glycosylation, underscoring the importance of molecular pathways beyond traditional bone turnover markers [17]. Metabolomics studies have revealed alterations in lipid, amino acid, and microbially derived metabolite profiles in individuals with osteoporosis, providing molecular signatures linked to risk and pathogenesis. Gut-microbiome–serum-metabolome integration has identified novel axes influencing BMD and fracture susceptibility [18]. Single-cell RNA-seq and ATAC-seq studies have delineated osteoclastogenic and osteoblastogenic trajectories, as well as epigenetic regulators of cell fate in aging [17]. Comparative transcriptomic analyses of male and female osteoblasts have revealed sex-specific molecular signatures of osteoporosis risk [19].

Collectively, these diverse applications demonstrate that multi-omics approaches are feasible, highly informative, and transformative for musculoskeletal research. Nevertheless, many studies remain at the discovery or pilot stage, and translation into diagnostic panels or clinical workflows is still limited. Moreover, relatively few investigations directly address osteoporosis as a unified bone–muscle disorder, highlighting a gap in the literature and an opportunity for synthesis and future research.

In this review, we aim to comprehensively examine the literature on multi-omics based diagnostics and early detection in osteoporosis. Specifically, our objectives are to: (i) map the spectrum of multi-omics technologies used in these conditions; (ii) catalog and evaluate molecular biomarkers and signatures proposed for early diagnosis, risk stratification, or disease subtyping; (iii) explore how multi-omics data elucidate the biology of bone–muscle crosstalk and the integrated pathophysiology of osteoporosis; and (iv) discuss translational challenges and future directions for implementing multi-omics in clinical practice for musculoskeletal aging disorders.

## 2. Methods

### 2.1. Information Sources and Search Strategy

This scoping review was conducted to identify studies employing multi-omics technologies—including genomics, transcriptomics, proteomics, metabolomics, and microbiomics—in the investigation of osteoporosis, sarcopenia, and the combined condition of osteoporosis. The review aimed to map current evidence on how multi-omics-based approaches contribute to understanding the molecular mechanisms underlying bone and muscle degeneration, as well as their potential applications in early diagnosis, personalized medicine, and therapeutic target discovery. Comprehensive searches were performed across three major scientific databases: PubMed, Embase, and Scopus. Search terms were combined using Boolean operators and included permutations of keywords such as “osteoporosis, “osteosarcopenia,” and “omics”. The search was completed on 15 January 2026 and therefore includes all articles published up to this date.

The eligibility criteria were defined according to the PCC (Population–Concept–Context) framework recommended for scoping reviews by the Joanna Briggs Institute [20]. Population (P): Studies involving human participants diagnosed with osteoporosis or osteosarcopenia, or at risk of bone decline in the context of osteoporosis-related conditions, were considered eligible. Concept (C): The review focused on the application of omics-based approaches, including but not limited to genomics (e.g., genome-wide association studies), transcriptomics, proteomics, metabolomics, epigenomics, and microbiome profiling. Eligible studies explored molecular biomarkers, pathogenic pathways, therapeutic targets, or personalized prevention and treatment strategies related to bone health or osteosarcopenia. Context (C): Studies conducted in clinical, epidemiological, or translational research settings were included without restriction on healthcare level or geographic location.

The review process adhered to the Preferred Reporting Items for Systematic Reviews and Meta-Analyses extension for Scoping Reviews (PRISMA-ScR) guidelines. The corresponding PRISMA-ScR checklist is provided in Supplementary File S1.

### 2.2. Eligibility Criteria

All records retrieved from the database searches were merged into a single dataset, and duplicate entries were removed. Then, independently, three authors screened each remaining article based on the following inclusion criteria: (i) original studies published in English; (ii) use of multi-omics technologies to investigate bone, muscle, or bone–muscle interactions in the context of osteoporosis or osteosarcopenia; (iii) Human studies or translational research with clear clinical relevance. Exclusion criteria included: (i) articles missing one or more keywords; (ii) book chapters, editorials, letters, or notes; (iii) conference abstract or paper; (iv) irrelevance to the main topic or lacking translational significance; (v) non-human studies; (vi) not open access; (vii) review, systematic review, or meta-analysis. Titles and abstracts were independently reviewed by three researchers, and any discrepancies were resolved through discussion with a fourth reviewer. The reasons for the exclusion of each record are reported in Supplementary File S2.

### 2.3. Data Extraction and Quality Process

Following selection, data extraction was carried out by manual curation. The data were extracted by three authors, who then independently summarized each article’s findings.

### 2.4. Methodological Approach to the Critical Appraisal

In line with the methodological framework of scoping reviews, a formal risk-of-bias assessment or quantitative quality scoring was not performed [20]. Instead, a descriptive critical appraisal of the included studies was conducted to provide an overview of methodological characteristics and research robustness across the field. The evaluation focused on key elements such as study design, sample size, population characteristics, integration of multi-omics approaches, validation strategies (including replication cohorts and functional assays), and level of clinical translation. This qualitative synthesis aimed to highlight methodological strengths, recurring limitations, and research gaps, without excluding studies based on quality criteria. The purpose of this appraisal was not to rank studies, but to contextualize the current state of evidence and inform directions for future research.

## 3. Results

### 3.1. Literature Research

**Figure 1:**
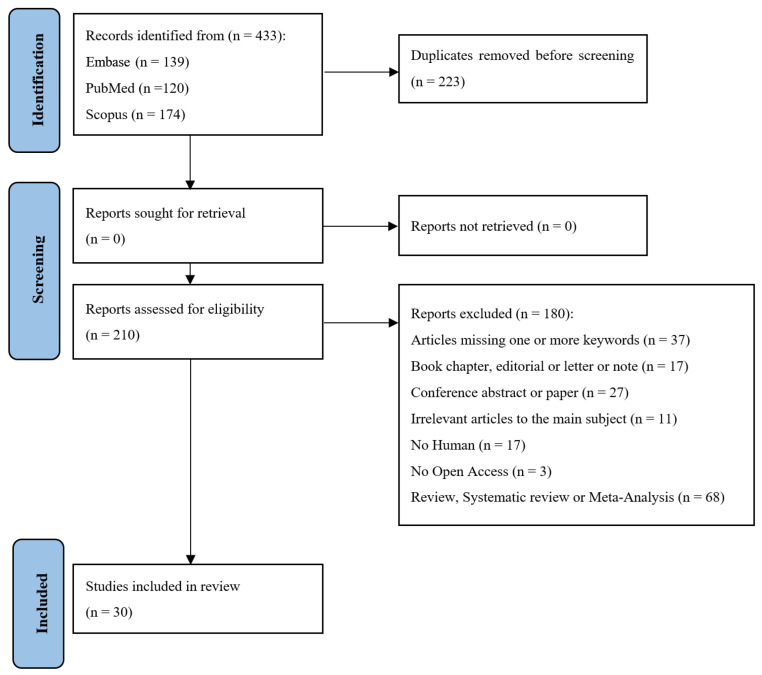
PRISMA-ScR flow-diagram showing research strategy [21].

The flowchart in Figure 1 provides an overview of the literature search and selection process. A total of 433 records were retrieved from the PubMed, Embase, and Scopus databases using combinations of the following keywords: “osteoporosis”, “osteosarcopenia”, and “omics”. After the removal of 223 duplicates, 211 unique articles were screened based on titles and abstracts according to the predefined inclusion and exclusion criteria. Following a detailed full-text assessment, 30 studies met the eligibility criteria and were included in this scoping review. These publications collectively illustrate the growing application of multi-omics technologies in elucidating the molecular landscape of bone and muscle degeneration. The final dataset provides a comprehensive foundation for synthesizing current evidence on how high-throughput molecular profiling contributes to understanding gene variants, epigenetic regulation, and metabolic pathways involved in bone loss and muscle atrophy. Moreover, it supports the interpretation of system-level insights into the bone–muscle–immune–microbiome axis, emphasizing the potential of multi-omics-based biomarkers for early detection, patient stratification, and therapeutic target discovery in osteoporosis.

### 3.2. Study Characteristics

The reviewed studies covered diverse disease stages, including early-life osteoporosis risk, osteopenia, post-menopausal osteoporosis, sarcopenia, age-related degeneration, and comorbid conditions such as type 1 diabetes and atherosclerosis (Table 1) [22,23,24,25,26].

Biological samples included plasma, serum, stool, bone and skeletal muscle tissue, bone marrow, tibia, lumbar spine, femoral neck, maternal and fetal specimens, and peripheral blood monocytes [27,28,29,30]. Study populations ranged from single cases to over one million participants [31,32]. Various omics approaches were employed, such as transcriptomics, proteomics, metabolomics, lipidomics, metagenomics, methylomics, epigenomics, and “oculomics” [23,28,33]. Integration levels varied, from single-omics analyses to high multi-omics integration combining scRNA-seq, bulk transcriptomics, WGCNA, Mendelian randomization (MR), machine learning (LASSO, Random Forest), Deep Latent Space Fusion (DLSF), and network or pathway analyses [28,33,34,35]. Several studies linked molecular profiles to bone mineral density (BMD) or osteoporosis-related phenotypes, while others focused on biomarker discovery or subtype stratification [28,36,37].

Overall, the studies highlighted the heterogeneity of experimental designs, populations, and analytical frameworks, reflecting the complex molecular landscape of bone- and muscle-related disorders.

**Table 1:** Overview of selected omics studies in osteoporosis.

Authors	Disease Stage	Biological Sample	Population	Type of Omics	Level of Integration
Chang S et al., 2025 [27]	Osteoporosis	Plasma	45 Osteoporosis 18 Controls	Transcriptomics Metabolomics	High integration: miRNA→gene networks →metabolites mapped in osteoporosis.
Chen J et al., 2024 [24]	Osteoporosis Sarcopenia Age-related degeneration	Skeletal muscle Tissue Bone Tissue	3 Osteosarcopenia 3 Osteoporosis	Transcriptomics Proteomics	Multi-tissue integration: bone + muscle, joint molecular profiling.
Choi JY et al., 2024 [23]	Osteopenia Osteoporosis	Lumbar Spine Femoral Neck	17,306 Osteoporosis	Oculomics	Single-dataset predictive modeling.
Eisfeldt J et al., 2022 [38]	Autism Epilepsy Osteoporosis	Neuroepithelial stem cells	1 Osteoporosis	Genomics Transcriptomics	Multi-omics: structural genome → disrupted gene → transcriptome changes; integration across genome + functional transcriptome + pathway analysis.
Fuzhu T et al., 2025 [34]	Post-menopausal Osteoporosis	Online Dataset	70 Post-menopausal 63 Controls	Transcriptomics	Multi-omics + computational integration: they combine scRNA-seq and bulk transcriptome data, apply differential expression, Mendelian randomization (MR), machine learning to build a diagnostic model; immune-infiltration analysis.
Greenbaum J et al., 2022 [28]	Post-menopausal Osteoporosis	Stool Serum	499 Post-menopausal	Metagenomics Metabolomics	Integration between microbiome → serum metabolome → bone mineral density (BMD) phenotype—moderate to high integration across omics and phenotypic outcome.
Guo J et al., 2022 [37]	Osteoporosis	Online Dataset	76 Osteoporosis 101 Controls	Transcriptomics	Stratification of patients into subtypes based on gene expression (lipid/steroid metabolism subtype, glycolysis subtype, polysaccharide subtype)—single omics but subtype integration.
Lan K et al., 2025 [31]	Osteoporosis	Online Dataset	2 Osteoporosis 2 Controls	Transcriptomics	High: gene expression (bulk and scRNA) → hub gene identification (CHAD, COL2A1).
Li C et al., 2023 [39]	Postmenopausal osteoporosis	Serum	46 Post-menopausal 42 Controls	Proteomics	Single-omics (proteome) focused on biomarker discovery.
Li M et al., 2024 [40]	Osteoporosis	Online Dataset	2 Osteoporosis	Transcriptomics	High integration: macrophage gene modules → ferroptosis pathway → bone microstructure deterioration.
Li Q et al., 2025 [41]	Osteoporosis	Online Dataset Bone marrow Femoral head	n.a.	Transcriptomics	Multi-omics integration: they integrated bulk + single-cell transcriptomics, used WGCNA (gene co-expression network), machine learning (neural nets) to identify hub genes, protein–protein interaction networks, and cellular communication inference.
Li Q et al., 2025 [42]	Osteoporosis	n.a.	n.a.	n.a.	Integration across gut microbiota and host immune system/gene expression.
Li YR et al., 2025 [43]	Osteoporosis	Online Dataset	n.a.	Transcriptomics Genomics	High integration: epidemiology + transcriptomics + machine learning + molecular modeling.
Lian J et al., 2025 [44]	Osteoporosis Sarcopenia Age-related degeneration	Femoral head Bone marrow	5 Osteoporosis 4 Controls	Transcriptomics	High integration: transcriptomic → eQTL/pQTL → Mendelian Randomization causal inference.
Ma C et al., 2025 [30]	Developmental/early life (prenatal exposure) → reduced peak bone mass → osteoporosis risk	Maternal fecal sample Maternal and fetal serum Fetal bone tissue Bone marrow mesenchymal stem cells	25 Pregnant	Metabolomics Transcriptomics Epigenomics	High integration: maternal microbiome → metabolite (daidzein) → offspring transcriptome/epigenome → osteogenic phenotypes.
Mishra BH et al., 2021 [26]	Subclinical disease stage Osteoporosis or Atherosclerosis	Serum Radius/Tibia Carotid intima-media thickness	1494 Osteoporosis	Lipidomics	Moderate to high integration: large lipidome dataset → statistical network analysis to identify modules associated jointly with subclinical osteoporosis and atherosclerosis markers.
Pontes TA et al., 2019 [36]	Established disease stage: postmenopausal women with osteopenia vs. osteoporosis	Serum Plasma	26 Osteopenia 24 Osteoporosis 28 Controls	Metabonomics	Moderate integration: metabolomic profiling + grouping by BMD/osteopenia/osteoporosis phenotype.
Qiu C et al., 2020 [33]	Osteoporosis	Serum	61 High BMD 58 Low BMD	Genomics Transcriptomics Methylomics Metabolomics	High integration: single-omics initial, then integrative canonical correlation analysis (SMDCCA), followed by QTL linking and Mendelian randomization (MR) causal inference.
Su K et al., 2024 [32]	Osteoporosis	Online Dataset	4982 Controls	Genomics	Moderately high: integration of SV detection, association with bone mineral density at multiple skeletal sites + co-occurrence with multi-omics.
Sun C et al., 2025 [45]	Osteoporosis	Online Dataset	9046 Osteoporosis 2085 Osteoporosis with pathological fracture 1709 Postmenopausal osteoporosis with pathological fracture 1.023,862 Controls	Genomics Transcriptomics	High integration: druggable gene list MR to test causal effect on osteoporosis phenotypes mediation pathways + drug prediction.
Tan Z et al., 2024 [29]	Osteoporosis	Tibia	243 Osteoporosis	Proteomics Transcriptomics	High: Integration of genetic variant → proteomics → single-cell transcriptomic trajectories → clinical phenotypes.
Wang H et al., 2025 [25]	Type 1 diabetes comorbid with osteoporosis	Online Dataset	63 Type 1 diabetes 52 Osteoporosis 116 Controls	Transcriptomics	Moderate–high: integrated DEGs from two diseases + autophagy-gene intersection + machine learning (LASSO/RF) + miRNA network.
Wen B et al., 2024 [46]	Osteoporosis	Peripheral blood Vertebral bone samples	500 Osteoporosis 500 Controls	Genomics Epigenomics	High: integrated genotype data + methylation profiling + clinical outcome (refracture) prediction.
Yuan C et al., 2024 [35]	Osteoporosis	Blood	532 Osteoporosis	Genomics	Very high integration: used a “Deep Latent Space Fusion” (DLSF) model to fuse multi-modal molecular signatures (M3S) from multi-omics + longitudinal data.
Zhang B et al., 2024 [47]	Osteoporosis	Plasma	5 Osteoporosis 5 Controls	Transcriptomics	High: Used single-cell annotation, pseudotime, machine-learning to integrate transcriptome + scRNA-seq + immune cell profiling.
Zhang C et al., 2019 [48]	Postmenopausal osteoporosis	Online Dataset	10 High BMD 10 Low BMD	Genomics Metabolomics	Moderate to high: Integrated six network types into one composite network to prioritize metabolites.
Zhang R et al., 2025 [49]	Osteoporosis	Online Dataset	1351 Osteoporosis 209,313 Controls	Transcriptomics Genomics	High: Integration of expression/methylation QTLs + GWAS + causal inference + colocalization.
Zhang ZL et al., 2024 [50]	Osteoporosis	Online Dataset	n.a.	Genomics Epigenomics	High: Two-sample lipid traits → methylation sites as mediators.
Zhao X et al., 2025 [51]	Osteopenia Osteoporosis	Online Dataset	20 Osteopenia 12 Osteoporosis 19 Controls	Metagenomics Metabolomics	High: Microbiome + metabolome data integrated, correlation networks, biomarker model.
Zhu W et al., 2017 [52]	Osteoporosis	Online Dataset	29 low hip BMD 30 high hip BMD	Proteomics	Moderate: Proteome profiling in monocytes, comparison low vs. high BMD; results linked to transcriptomic/genomic evidence.

Abbreviation: BMD: Bone Mineral Density; DLSF: Deep Latent Space Fusion; DEGs: Differentially Expressed Genes; eQTL: expression Quantitative Trait Loci; GWAS: Genome-Wide Association Study; LASSO: Least Absolute Shrinkage and Selection Operator; M3S: Multi-Modal Molecular Signatures; MR: Mendelian randomization; n.a.: not available/non applicable; pQTL: protein Quantitative Trait Loci; QTL: Quantitative Trait Loci; RF: Random Forest; scRNA-seq: single-cell RNA sequencing; WGCNA: Weighted Gene Co-expression Network Analysis.

### 3.3. Multi-Omics Insights into Osteoporosis

Recent multi-omics studies have substantially refined our understanding of osteoporosis by moving beyond single-layer associations toward integrated biological patterns that link molecular mechanisms, tissue crosstalk, and clinical heterogeneity [24,27,28,30,31,33,35,37,40,41,51,52] (Table 2). Across transcriptomic, proteomic, metabolomic, epigenomic, and microbiome datasets, a consistent convergence emerges on pathways related to bone remodeling, immune regulation, cellular metabolism, and ferroptosis, highlighting shared molecular drivers of disease across omics layers [24,27,31,41]. A recurring theme across studies is the integration of immune and bone pathways, with NF-κB signaling, osteoclast differentiation, macrophage activation, and inflammatory mediators repeatedly identified across transcriptomic and proteomic analyses [24,27,41]. These immune–bone interactions are closely intertwined with metabolic reprogramming, as lipid, amino acid, and energy metabolism signatures influencing bone turnover and fracture risk have been reported consistently across metabolomic and integrated multi-omics studies [27,33,37,51]. Multi-omics analyses also emphasize the bone–muscle axis, revealing shared molecular signatures underlying concurrent bone loss and muscle degeneration. Integrated transcriptomic and proteomic approaches have identified cytoskeletal, mitochondrial, and inflammatory pathways linking osteoporosis and sarcopenia, supporting the concept of a unified musculoskeletal aging process [24,31].

Differences among omics modalities further refine disease characterization. While transcriptomic and epigenomic analyses predominantly capture upstream regulatory and inflammatory processes [33,51], metabolomic and microbiome-based studies provide functional insights into systemic metabolism and host–microbe interactions. In particular, the gut–bone axis has emerged as a reproducible theme, with microbiome–metabolite crosstalk offering non-invasive biomarkers and potential preventive strategies [28,52]. Patient stratification represents another major advance enabled by multi-omics integration. Distinct molecular subtypes of osteoporosis, defined by metabolic and inflammatory signatures, have been associated with differential clinical phenotypes and responses to interventions such as calcium supplementation [37]. These findings underscore the promise of precision medicine approaches, although their clinical translation remains limited by cohort size and population heterogeneity.

Finally, longitudinal and multi-layer studies integrating genetic, epigenetic, and metabolomic data suggest that early-life and developmental exposure shapes peak bone mass and long-term osteoporosis risk through persistent inflammatory and epigenetic programming [30,33,35,51]. Despite these advances, important research gaps remain, including inconsistencies across omics platforms, limited functional validation of candidate targets [31,40,41], and a lack of large, harmonized cohorts. Addressing these challenges will be essential to translate multi-omics discoveries into robust biomarkers and targeted therapies for osteoporosis.

**Table 2:** Summary of molecular findings and translational relevance in bone–muscle disorders.

Authors	Disease Stage	Main Findings	Potential for Clinical Application	Methodological Quality
Chang S et al., 2025 [27]	Osteoporosis	Identified metabolic-related genes and metabolites; built regulatory network for osteoporosis metabolism.	Biomarkers with better diagnostic performance than traditional bone markers; potential early detection tool.	Moderate sample size; single-site; further validation needed for wide clinical application.
Chen J et al., 2024 [24]	Osteoporosis Sarcopenia Age-Related degeneration	Discovered genes/proteins (e.g., PDIA5, TUBB1, MYH7) linked to bone and muscle degeneration; highlighted osteoclast differentiation, NF-κB signaling pathways.	Offers targets for preventing/treating combined bone-muscle loss; improved stratification of older patients.	Good integration; tissue-based; likely moderate sample size.
Choi JY et al., 2024 [23]	Osteopenia Osteoporosis	Develop and validate risk prediction models for osteopenia/ osteoporosis using demographic, anthropometric, exam and ophthalmologic variables.	Risk stratification tool for early screening in general population.	Large, nationally representative sample; cross-sectional design; uses DXA; limited to available variables; prediction model rather than mechanistic multi-omics.
Eisfeldt J et al., 2022 [38]	Autism Epilepsy Osteoporosis	Identified disruption of gene MINK1 by the translocation; in patient neuroepithelial stem cells, MINK1 expression reduced >50% vs. controls; differentially expressed 539 genes; enrichment of ossification and nervous system-development pathways.	Potential diagnostic/monogenic gene identification in rare cases; shows utility of long-read genome sequencing and transcriptome in clinical genetics diagnostics.	Methodologically robust for a case study: used multiple genome sequencing technologies (short, linked, long-read) + optical mapping; derived patient iPSCs → NESCs for functional assay; but n = 1 (single patient) so generalizability limited.
Fuzhu T et al., 2025 [34]	Post-menopausal Osteoporosis	Identified lactylation-related genes (e.g., CSRP2, FUBP1) as biomarkers; nomogram for early prediction of osteoporosis risk.	Useful for early risk stratification in post-menopausal women; may guide preventive strategies.	Emerging study; marker discovery phase; requires larger validation cohorts.
Greenbaum J et al., 2022 [28]	Osteoporosis	Identified 22 bacterial species and 17 metabolites associated with BMD; constructed inter-omics network showing microbiome–metabolite crosstalk relevant to skeletal remodeling.	Novel biomarkers or mechanistic insights into bone health via gut-bone axis; potential for early risk stratification or preventive interventions.	Large sample size; cross-sectional, exploratory; associations not yet validated or causal; FDR correction limited significance.
Guo J et al., 2022 [37]	Osteoporosis	Identified three distinct metabolism-related gene subtypes in osteoporosis and 10 characteristic genes (e.g., GPR31, GATM, DDB2…) that may relate to metabolic pathogenesis.	May support patient stratification and design of metabolism-targeted interventions in osteoporosis; biomarker research direction.	Good genomic analysis; sample size and external validation unclear; more functional/mechanistic work needed.
Lan K et al., 2025 [31]	Osteoporosis	Identified CHAD and COL2A1 as down-regulated in OP; docking showed wogonin/tetrandrine high affinity with CHAD/COL2A1; in vitro wogonin enhanced chondrogenic differentiation of ATDC5 cells.	Suggests new gene target (CHAD) and compound (wogonin) for OP, possibly via cartilage/chondrocyte axis of bone health.	Rigorous bioinformatics + in vitro work; still preclinical; gene/transcriptome focused not yet in large clinical cohorts.
Li C et al., 2023 [39]	Postmenopausal osteoporosis	Identified serum proteins (e.g., CDH1 up, PNP down) with good diagnostic sensitivity.	Potential non-invasive biomarkers for PMOP diagnosis and prediction.	Relatively small discovery sample; strong follow-up validation; proteomic only (no multi-omics); needs further large-scale validation.
Li M et al., 2024 [40]	Osteoporosis	Identified 12 BM-MSC subsets with distinct distributions; key LR pairs (MIF-CD74, ITGB2-ICAM2) linked to immune score; CD74 identified as a target; 48 drugs targeting CD47/CD74 were screened, with DB01940 showing strong binding.	Provides a framework for drug repurposing in osteoporosis; identifies actionable target (CD74) and candidate drug(s) for further testing.	Strong integrative analysis; human data; still in silico/preclinical phase for many drug candidates; validation in clinical trials needed.
Li Q et al., 2025 [41]	Osteoporosis	Identified 1705 macrophage marker genes and 839 module genes; ferroptosis pathway enrichment; SMAD7 hub gene; validation: inhibition of SMAD7 (via Mongersen) attenuated macrophage ferroptosis and improved bone microstructure.	Suggests SMAD7 as novel therapeutic target in osteoporosis via macrophage ferroptosis.	Robust mechanistic and multi-omics work; translational step still required; more human cohort validation needed.
Li Q et al., 2025 [42]	Osteoporosis	Developed a novel integrative statistical method (sparse group multitask regression) for combining diverse omics datasets; applied it to osteoporosis/BMD studies; identified 7 significantly associated genes (e.g., SOD2, TREML2, HTR1E, GLO1).	The method provides an approach to identify risk genes for osteoporosis that may be otherwise missed by standard meta-analysis; could help in biomarker discovery.	Good methodological innovation; real-data application to osteoporosis; limitations include relatively modest sample sizes in expression cohorts, integration only SNP + mRNA data, no direct translational therapeutic validation.
Li YR et al., 2025 [43]	Osteoporosis	Cadmium exposure identified as risk factor; highlighted genes FOXO3, CCND1, MAP1LC3B, HMOX1, MT1G; HMOX1 linked to M2 macrophage polarization; geniposide identified as potential ligand for HMOX1.	Suggests HMOX1 as therapeutic target in cadmium-induced bone damage; supports exposure-based prevention.	Innovative integration; cross-sectional design limits causal inference; MR helps but exposure measurement may have limitations; needs prospective/interventional follow-up.
Lian J et al., 2025 [44]	Osteoporosis Sarcopenia Age-related degeneration	Identified CPXM1 as causally associated with increased osteoporosis risk; suggested ECM degradation/impaired osteoblast differentiation pathways.	CPXM1 proposed as a novel drug target; predicted repurposing candidates.	Strong multi-omics causal design; limited by small human sample; population ancestry limited; translational gap acknowledged.
Ma C et al., 2025 [30]	Developmental/early life (prenatal exposure) → reduced peak bone mass → osteoporosis risk	Prenatal prednisone exposure (PPE) alters maternal gut microbiota, depletes daidzein (DAI), leading to suppressed Hoxd12 expression, impaired osteogenesis and reduced peak bone mass in female offspring. Maternal DAI supplementation prevented these effects. PubMed + 1	Maternal DAI supplementation during pregnancy may serve as a preventive strategy against offspring osteoporosis risk due to prenatal glucocorticoid exposure.	Strong experimental design: human + animal + multi-omics + mechanistic validation (cell, epigenetic) noted in full text. Limitations: translational leap to humans (supplementation in pregnancy); sex-specific effect (female only) needs broader validation.
Mishra BH et al., 2021 [26]	Subclinical disease stage Osteoporosis or Atherosclerosis	Identified a lipid-module (105 lipid species, mostly glycerolipids, glycerophospholipids, sphingolipids) jointly associated with subclinical osteoporosis and atherosclerosis.	The lipid module may serve as biomarker signature for comorbidity risk (osteoporosis + atherosclerosis), opening potential for dual-disease risk stratification or preventive strategies.	Large cohort, strong multi-omics profiling, good statistical network approach. Limitations: cross-sectional/associative (not necessarily causal); limited to subclinical surrogate markers; need further validation and functional mechanistic work.
Pontes TA 2019 et al., [36]	Established disease stage: postmenopausal women with osteopenia vs osteoporosis	^1H NMR metabonomics could discriminate between osteopenia and osteoporosis in postmenopausal women; identified metabolites associated with disease-stage difference.	Potential use in clinical diagnosis/staging of bone health in postmenopausal women; metabonomic biomarker panels to differentiate osteopenia vs. osteoporosis.	Good proof-of-concept; modest sample size; only metabolomics layer; no downstream clinical trial or functional validation; may need larger and longitudinal studies.
Qiu C et al., 2020 [33]	Osteoporosis	Identified an optimal multi-omics biomarker panel (74 DEGs, 75 methylation sites, 23 metabolites). Found 199 QTLs connecting these biomarkers with genetic variants. Network/pathway analysis showed enrichment in bone-related pathways (RANK/RANKL, MAPK/TGF-β, WNT/β-catenin). Five biomarkers (FADS2, ADRA2A, FMN1, RABL2A, SPRY1) showed causal effect on BMD via MR.	Potential for biomarker development (diagnostic/predictive) for osteoporosis risk; provides mechanistic insight to guide prevention or therapeutic stratification.	Strong human multi-omics study with causal MR analysis; limitations: sample size moderate (119); only females; ethnic/caucasian limitation; follow-up functional validation limited; biomarkers still early stage for clinical translation.
Su K et al., 2024 [32]	Osteoporosis	Identified significant SV-BMD associations (125 for femoral neck, 99 for spine, 83 for hip) explaining ~13.3–19.1% of BMD variance. Novel genes prioritized: LINC02370, ZNF family, ZDHHC family, FMN2, LINC00494, IBSP, SPP1.	Provides new genetic targets/regions for osteoporosis risk prediction; possible novel therapeutic/biomarker targets.	Large sample, multi-ethnic, high-quality WGS and SV analysis; strengths: multi-site BMD, stratification by sex/ethnicity; limitations: still association only, SV functional validation needed; age relatively young (~39) so pre-osteoporosis rather than overt disease.
Sun C et al., 2025 [45]	Osteoporosis	Identified three potential therapeutic targets for osteoporosis: TAS1R3, TMX2, and SREBF1. Pathways identified include lipid metabolism, immune expression, insulin resistance. Phe-MR suggests associations.	Offers candidate targets for drug development or repositioning in osteoporosis; may inform precision therapies.	Strong methodological design (MR + multi-omics + druggable target focus); limitations: those are candidates not yet clinically tested; observational genetic inference; need functional/clinical validation.
Tan Z et al., 2024 [29]	Osteoporosis	Identified lactylation-related gene markers (e.g., CSRP2 downregulated, FUBP1 upregulated) associated with risk of osteoporosis.	Potential for early prediction/diagnostic biomarkers in PMOP; might lead to targeted therapies/modulation of lactylation pathways.	Relatively new area (lactylation in bone); details limited; needs validation in larger and diverse cohorts; mechanistic work pending.
Wang H et al., 2025 [25]	Genetic skeletal disease (type XV OI) Osteoporosis	Found that WNT1 loss-of-function causes impaired secretion/activity → porous bone structure, altered cellular differentiation trajectories (excess CXCL12+ progenitors, fewer mature osteocytes) and increased osteoclastic activity	Provides mechanistic insight into WNT1’s role in bone cell differentiation; may inform therapeutic strategies for OI and related low-bone-mass conditions (including early onset osteoporosis)	Strong study: human patients with rare variant, multi-omics + cellular assays; limitation: specific rare disease context (OI) rather than general osteoporosis; translation to common OP may need caution
Wen B et al., 2024 [46]	Osteoporosis	Identified 21 autophagy-related hub genes common to T1DM and OP (e.g., CPNE1, FRAT2) via machine learning; implicated Wnt, immune infiltration and autophagy pathways.	CPNE1 and FRAT2 proposed as biomarkers/targets for dual T1DM-OP intervention; opens path for targeted therapy of the comorbidity.	Strong design in silico; limitation: purely bioinformatics (no new patient validation or functional in vitro/in vivo experiments).
Yuan C et al., 2024 [35]	Osteoporosis	Identified two clinically relevant osteoporosis sub-types (CISs) in Chinese individuals, which differed in bone mineral density response to calcium supplementation after 2-year follow-up, and in fracture risk at 4-year follow-up.	The multi-omic subtype classification may allow more precise risk stratification (which patients benefit from supplementation) and inform tailored intervention strategies.	Strong design: large cohort, longitudinal, multi-omics, validation cohort; Limitations: ethnic/racial generalizability to non-Chinese populations may be limited; full list of multi-omics layers and effect sizes may need further detail
Zhang B et al., 2024 [47]	Osteoporosis	Found four hub immune-related genes (DND1, HIRA, SH3GLB2, F7) that are reduced in OP; neutrophils and BM-MSC proportions were increased in OP; indicates immune cell involvement in OP.	These hub genes could act as immune-biomarkers for OP; immune-modulatory therapies might be explored.	Good mechanistic and biomarker study; limitations: small validation sample (n = 5 + 5), mostly computational; further functional/clinical validation required.
Zhang C et al., 2019 [48]	Postmenopausal osteoporosis	Prioritized top 50 candidate metabolites for PMO; top 5 included glucosylgalactosyl hydroxylysine, all-trans-5,6-epoxyretinoic acid, tretinoin, colecalciferol, rocaltrol. Tretinoin and estraderm flagged as especially relevant.	Provides candidate metabolite biomarkers/therapeutic leads for PMO; could guide metabolomics-based diagnostics or interventions.	Computational network approach; limitations: small sample size (n = 20), no experimental/clinical validation of metabolites; exploratory rather than confirmatory.
Zhang R et al., 2025 [49]	Osteoporosis	Identified two IR-genes causally linked: increased expression of FAS → increased OP risk; increased expression of CHUK → decreased OP risk. Colocalization indicated interactions with hormones/inflammatory factors (e.g., estradiol, IL-1α).	Highlights inflammatory-gene targets for OP prevention/therapy; opens path to immune-modulatory approaches.	Good multi-omics MR design; limitations: still gene-level only, functional/clinical validation needed; as typical of MR, only infers causality under assumptions.
Zhang ZL et al., 2024 [50]	Osteoporosis	Found negative causal associations of lipid traits (e.g., LDL-C, VLDL-C, HDL-C) with BMD; identified 3 methylation sites (cg15707428 in GREB1; cg16000331 in SREBF2; cg14364472 in NOTCH1) linking lipid genetic effects to BMD.	Provides insight into lipid-metabolism → methylation → bone health axis and identifies epigenetic biomarkers/targets for osteoporosis related to dyslipidemia.	Strong design using MR and epigenetic mediation; limitations: observational genetic inference, need functional/clinical follow-up, BMD not always fracture outcomes.
Zhao X et al., 2025 [51]	Osteopenia Osteoporosis	Found distinct gut microbial/metabolic signatures in low bone mass patients. Notably, enrichment of Lachnospira eligens in low bone mass group, depletion of beneficial taxa (e.g., Bifidobacterium, Bacteroides stercoris). Identified 127 differential metabolites; built a 4-species microbial model with AUC >0.9 for LBM vs control.	Non-invasive biomarkers (microbiome and metabolite signatures) for assessing bone health in fracture patients; potential for gut-bone axis-based interventions.	Strong, novel design (human fracture patients, multi-omics). Limitations: cross-sectional (not longitudinal); modest sample size; mechanistic causality not demonstrated; needs external validation.
Zhu W et al., 2017 [52]	Osteoporosis	Detected ~3796 cytosolic proteins; identified 16 significant and 22 suggestive DEPs between low and high BMD. Highlighted proteins/genes ALDOA, MYH14, Rap1B as associated with BMD and likely monocyte-mediated mechanisms.	Potential biomarkers in monocyte proteome for male osteoporosis; insights into monocyte/osteoclast pathway regulation in bone loss.	Innovative subcellular proteomics in monocytes; limitations: male only, BMD low vs. high (not fracture outcome), no direct functional experiments for all identified proteins.

Abbreviations: ^1H NMR: proton nuclear magnetic resonance; AUC: area under the curve; BMD: bone mineral density; BM-MSCs: bone marrow mesenchymal stem cells; CISs: clinically identified subtypes; DEGs: differentially expressed genes; DEPs: differentially expressed proteins; ECM: extracellular matrix; FDR: false discovery rate; IR genes: immune-related genes; LBM: low bone mass; LR: ligand–receptor; MR: Mendelian randomization; OI: osteogenesis imperfecta; OP: osteoporosis; Phe-MR: phenome-wide Mendelian randomization; PMOP: postmenopausal osteoporosis; QTLs: quantitative trait loci; SNP: single nucleotide polymorphism; SVs: structural variants; T1DM: type 1 diabetes mellitus; WGS: whole-genome sequencing.

### 3.4. Critical Evaluation of Research Quality

The body of research in osteoporosis and musculoskeletal aging demonstrates a wide spectrum of methodological quality, ranging from small exploratory studies to large, multi-omics, and population-based cohorts [27,32,35,37]. Several studies are characterized by moderate or modest sample sizes and single-site designs, often requiring further validation before wide clinical application [29,31,36,39]. Others have achieved good integration of tissue-based or human data, although sample sizes remain limited or validation in independent cohorts is pending [24,29,40]. Large, nationally representative cross-sectional studies employing DXA provide valuable population-level insights but are often restricted to available variables and emphasize predictive modeling rather than mechanistic understanding [23,32]. Advanced mechanistic studies, including those using multiple genome sequencing technologies and iPSC-derived NESCs for functional assays, demonstrate methodological rigor, but their generalizability is constrained by very small sample sizes, often n = 1 [38]. Emerging studies in marker discovery, lactylation, or metabolomics layers similarly highlight the need for larger, diverse cohorts and translational validation [34,36,53]. Some studies employ multi-omics approaches, including proteomic, transcriptomic, epigenetic, and network analyses, demonstrating strong integrative or causal designs (e.g., Mendelian randomization), yet functional validation and clinical translation remain limited, with many still in silico or preclinical [33,44,46,50].

Large-scale, longitudinal cohorts and multi-ethnic WGS studies offer high-quality data with stratification by sex and ancestry, but limitations include cross-sectional design, subclinical surrogate markers, and the early stage of biomarker or drug target translation [32,35,45]. Several studies focus exclusively on female populations or rare disease contexts, which restricts generalizability [29,30,46]. Computational network analyses and exploratory metabolomics studies provide hypothesis-generating insights but require experimental and clinical follow-up [26,48,49]. Overall, despite innovative designs and strong methodological frameworks, the field continues to face challenges regarding sample size, ethnic diversity, mechanistic validation, and translational applicability, underscoring the need for large, multi-center, longitudinal studies that integrate multi-omics, clinical phenotypes, and functional assays.

## 4. Discussion

The findings of this scoping review demonstrate that multi-omics approaches are profoundly reshaping the understanding of osteoporosis by revealing its complex, multilayered molecular architecture. Rather than arising from isolated alterations in bone tissue, osteoporosis emerges as a systems-level disorder driven by coordinated dysregulation across genomic, transcriptomic, proteomic, metabolomic, epigenomic, and microbiome-related networks. Evidence synthesized from 30 eligible studies shows that integrating multiple omics layers enables the identification of biologically meaningful molecular endotypes that transcend BMD as a sole disease descriptor.

A central implication of the reviewed literature is the dominant role of transcriptomics as a foundational layer for molecular characterization and patient stratification in osteoporosis. Several studies demonstrated that gene-expression profiles can delineate biologically distinct metabolic subtypes, including glycolysis-, lipid/steroid-, and polysaccharide-driven phenotypes [37]. High levels of integration were achieved when transcriptomics was combined with other omics layers. Chang et al. [27] exemplified this approach by integrating transcriptomics and metabolomics to construct miRNA–gene–metabolite networks regulating osteoporosis-related metabolic pathways. Similarly, Lan et al. [31] and Zhang B et al. [48] extended transcriptomic analyses to single-cell resolution, identifying hub genes and immune-related trajectories linked to bone loss.

Advanced computational and integrative transcriptomic pipelines further strengthened causal inference. Fuzhu et al. [34] bulk RNA-seq, scRNA-seq, Mendelian randomization, immune-infiltration analysis, and machine learning to construct a diagnostic framework for postmenopausal osteoporosis. Comparable high-dimensional approaches were reported by Li Q et al. [41] and Li M et al. [40], who linked macrophage gene modules and ferroptosis pathways to bone microstructural deterioration. These findings illustrate how transcriptomics-centered integration can uncover cellular drivers of disease that are amenable to experimental validation.

Genomics and epigenomics studies contributed essential architectural insights into osteoporosis susceptibility and fracture risk. Structural variation analyses revealed that copy-number and structural variants explain a substantial proportion of BMD variability across skeletal sites [32] while long-read sequencing highlighted how rare structural disruptions translate into transcriptomic and pathway-level changes relevant to bone fragility [38].

Large-scale Mendelian randomization and QTL colocalization studies further identified causal genes and regulatory mechanisms underlying osteoporosis phenotypes [47,48] direct clinical relevance were reported by Wen et al. [46] who identified methylation signatures predictive of refracture risk.

Multi-omics integrative studies provided some of the strongest evidence for causal biological pathways and therapeutic targets. Qiu et al. [33] combined genomics, transcriptomics, methylomics, and metabolomics using integrative correlation analyses and Mendelian randomization to identify causal biomarkers linked to BMD variation. Lian et al. [44] applied an eQTL/pQTL framework to identify CPXM1 as a potentially druggable target relevant to both osteoporosis and sarcopenia. Translational momentum was further demonstrated by Sun et al. [45], who integrated genomics and transcriptomics to identify druggable genes and infer therapeutic pathways across multiple osteoporosis-related phenotypes. Yuan et al. [35] presented one of the most advanced examples of machine-learning-based multi-omics fusion, leveraging deep latent space models to integrate longitudinal molecular signatures.

Proteomics, metabolomics, and lipidomics studies added functional depth by capturing downstream biochemical alterations associated with both early and established disease stages. Proteomic investigations identified circulating biomarkers with diagnostic potential [42] and immune-related proteomic signatures associated with BMD variation [52]. Integrative proteo-transcriptomic studies, such as those by Tan et al. [29] demonstrated how genetic variants propagate through protein networks and cellular differentiation trajectories, offering mechanistic insight applicable to both rare and common skeletal disorders. Metabolomic and lipidomic profiling further linked amino acids, glycerophospholipids, and lipid modules to subclinical and overt osteoporosis [26,36].

The gut–bone axis emerged as a recurrent and biologically consistent theme. Greenbaum et al. [28] demonstrated microbiome–metabolome–BMD integration in a large postmenopausal cohort, while Zhao et al. [51] constructed predictive models based on microbiome-derived metabolites distinguishing low bone mass from controls. Multi-generational implications were highlighted by Ma et al. [30], who showed that maternal microbiota-derived daidzein influences offspring bone development through coordinated metabolic, transcriptomic, and epigenomic regulation.

Several studies emphasized osteoporosis as a multisystem disorder involving bone–muscle and cross-disease interactions. Chen et al. [24] integrated transcriptomics and proteomics across bone and skeletal muscle, identifying shared molecular signatures relevant to osteosarcopenia. Cross-disease transcriptomic integration revealed shared autophagy-related pathways between osteoporosis and type 1 diabetes [25], while environmental genomics studies demonstrated how cadmium exposure modulates osteoporosis risk through gene–environment interactions [41]. Oculomics-based predictive modeling also illustrated how non-invasive phenotypic data can complement molecular stratification at scale [23].

Despite these advances, common limitations persist across the current omics literature. Many studies are constrained by small sample sizes, limited ethnic diversity, cross-sectional designs, and heterogeneous analytical pipelines. Functional validation remains uneven, with numerous candidate biomarkers and pathways lacking experimental confirmation. These limitations underscore the need for standardized methodologies and collaborative, multicenter study designs.

Overall, the synthesized evidence indicates that multi-omics approaches—from single-cell sequencing and microbiome profiling to Mendelian randomization and machine-learning fusion—are progressively decoding the complex molecular networks governing osteoporosis. The field is transitioning toward precision medicine, in which molecular endotypes, rather than BMD alone, may guide individualized prevention and treatment. Future progress will require large-scale, longitudinal, multi-omics cohorts integrated with clinical phenotyping and functional experimentation to validate biomarkers and enable the development of combined diagnostic algorithms and targeted therapeutic strategies specific to osteoporosis.

## 5. Conclusions

Overall, current research demonstrates the strong potential of multi-omics technologies to transform the understanding and clinical management of osteoporosis and related musculoskeletal disorders. Future progress will depend on large, multi-ethnic, longitudinal studies that integrate multi-omics data with clinical phenotypes and experimental validation. Such efforts will be essential to translate emerging biomarkers and molecular pathways into precision diagnostics and targeted interventions.

## Data Availability

All data generated or analyzed during this study are included in this published article and its Supplementary Information Files.

## Supplementary Materials

The following supporting information can be downloaded at https://www.mdpi.com/article/10.3390/cells15050472/s1, Supplementary File S1: PRISMA Checklist; Supplementary File S2: List of studies not included and exclusion reasons.

## Abbreviations

The following abbreviations are used in this manuscript:

BMD	Bone Mineral Density
CT	Computed Tomography
DEGs	Differentially Expressed Genes
DLSF	Deep Latent Space Fusion
DXA	Dual-Energy X-ray Absorptiometry
eQTL	Expression Quantitative Trait Locus/Loci
FRAX	Fracture Risk Assessment Tool
LASSO	Least Absolute Shrinkage and Selection Operator
M3S	Multi-Modal Molecular Signatures
MR	Mendelian Randomization
MRI	Magnetic Resonance Imaging
OI	Osteogenesis Imperfecta
PMOP	Postmenopausal Osteoporosis
pQTL	Protein Quantitative Trait Locus/Loci
QTL	Quantitative Trait Locus/Loci
RF	Random Forest
scRNA-seq	Single-Cell RNA Sequencing
SMDCCA	Sparse Multiple Discriminant Canonical Correlation Analysis
SV	Structural Variant
WGCNA	Weighted Gene Co-Expression Network Analysis
