# Supplementary material for: Profiling Osteoporosis via Integrated Multi-Omics Technologies
Source: Cells. 2026 Mar 5;15(5):472. doi: 10.3390/cells15050472 (PMC12984513; doi:10.3390/cells15050472)
Supplement: Supplementary file 1 [file cells-15-00472-s001.zip › cells-4162830-supplementary.pdf]

Supplementary File S1. Preferred Reporting Items for Systematic reviews and Meta-Analyses extension for Scoping Reviews (PRISMA-ScR) Checklist.

SECTION	ITEM	PRISMA-ScR CHECKLIST ITEM	REPORTED ON PAGE #
**TITLE**			
Title	1	Identify the report as a scoping review.	1
**ABSTRACT**			
Structured summary	2	Provide a structured summary that includes (as applicable): background, objectives, eligibility criteria, sources of evidence, charting methods, results, and conclusions that relate to the review questions and objectives.	1
**INTRODUCTION**			
Rationale	3	Describe the rationale for the review in the context of what is already known. Explain why the review questions/objectives lend themselves to a scoping review approach.	2-3
Objectives	4	Provide an explicit statement of the questions and objectives being addressed with reference to their key elements (e.g., population or participants, concepts, and context) or other relevant key elements used to conceptualize the review questions and/or objectives.	2-3
**METHODS**			
Protocol and registration	5	Indicate whether a review protocol exists; state if and where it can be accessed (e.g., a Web address); and if available, provide registration information, including the registration number.	Not applicable
Eligibility criteria	6	Specify characteristics of the sources of evidence used as eligibility criteria (e.g., years considered, language, and publication status), and provide a rationale.	3-4
Information sources*	7	Describe all information sources in the search (e.g., databases with dates of coverage and contact with authors to identify additional sources), as well as the date the most recent search was executed.	3-4
Search	8	Present the full electronic search strategy for at least 1 database, including any limits used, such that it could be repeated.	3-4
Selection of sources of evidence†	9	State the process for selecting sources of evidence (i.e., screening and eligibility) included in the scoping review.	3-4
Data charting process‡	10	Describe the methods of charting data from the included sources of evidence (e.g., calibrated forms or forms that have been tested by the team before their use, and whether data charting was done independently or in duplicate) and any processes for obtaining and confirming data from investigators.	3-4
Data items	11	List and define all variables for which data were sought and any assumptions and simplifications made.	3-4
Critical appraisal of individual sources of evidence§	12	If done, provide a rationale for conducting a critical appraisal of included sources of evidence; describe the methods used and how this information was used in any data synthesis (if appropriate).	3-4
Synthesis of results	13	Describe the methods of handling and summarizing the data that were charted.	3-4

SECTION	ITEM	PRISMA-ScR CHECKLIST ITEM	REPORTED ON PAGE #
**RESULTS**			
Selection of sources of evidence	14	Give numbers of sources of evidence screened, assessed for eligibility, and included in the review, with reasons for exclusions at each stage, ideally using a flow diagram.	5
Characteristics of sources of evidence	15	For each source of evidence, present characteristics for which data were charted and provide the citations.	6-10
Critical appraisal within sources of evidence	16	If done, present data on critical appraisal of included sources of evidence (see item 12).	17-18
Results of individual sources of evidence	17	For each included source of evidence, present the relevant data that were charted that relate to the review questions and objectives.	11-17
Synthesis of results	18	Summarize and/or present the charting results as they relate to the review questions and objectives.	
**DISCUSSION**			
Summary of evidence	19	Summarize the main results (including an overview of concepts, themes, and types of evidence available), link to the review questions and objectives, and consider the relevance to key groups.	18-19
Limitations	20	Discuss the limitations of the scoping review process.	20
Conclusions	21	Provide a general interpretation of the results with respect to the review questions and objectives, as well as potential implications and/or next steps.	20
**FUNDING**			
Funding	22	Describe sources of funding for the included sources of evidence, as well as sources of funding for the scoping review. Describe the role of the funders of the scoping review.	20

JBI = Joanna Briggs Institute; PRISMA-ScR = Preferred Reporting Items for Systematic reviews and Meta-Analyses extension for Scoping Reviews.

* Where *sources of evidence* (see second footnote) are compiled from, such as bibliographic databases, social media platforms, and Web sites.

† A more inclusive/heterogeneous term used to account for the different types of evidence or data sources (e.g., quantitative and/or qualitative research, expert opinion, and policy documents) that may be eligible in a scoping review as opposed to only studies. This is not to be confused with *information sources* (see first footnote).

‡ The frameworks by Arksey and O'Malley (6) and Levac and colleagues (7) and the JBI guidance (4, 5) refer to the process of data extraction in a scoping review as data charting.

§ The process of systematically examining research evidence to assess its validity, results, and relevance before using it to inform a decision. This term is used for items 12 and 19 instead of "risk of bias" (which is more applicable to systematic reviews of interventions) to include and acknowledge the various sources of evidence that may be used in a scoping review (e.g., quantitative and/or qualitative research, expert opinion, and policy document).

From: Tricco AC, Lillie E, Zarin W, O'Brien KK, Colquhoun H, Levac D, et al. PRISMA Extension for Scoping Reviews (PRISMAScR): Checklist and Explanation. Ann Intern Med. 2018;169:467–473. doi: 10.7326/M18-0850.

Supplementary File S2. List of articles excluded via eligible criteria application.

Authors		Title	Year	Exclusion Criteria
Abood A	et al	Using "-omics" Data to Inform Genome-wide Association Studies (GWASs) in the Osteoporosis Field	2021	Review
Ahmad SMS	et al	Molecular insights and emerging therapeutic perspectives of the lncRNA GAS5/miR-21 axis in cancer	2025	Review
Ahmed F	et al	A systematic review of computational approaches to understand cancer biology for informed drug repurposing	2023	Review
Alam MA	et al	A generalized kernel machine approach to identify higher-order composite effects in multi-view datasets	2021	Missing one or more keywords
Ashikov A	et al	Integrating glycomics and genomics uncovers SLC10A7 as essential factor for bone mineralization by regulating post-Golgi protein transport and glycosylation	2018	Irrelevant
Bencharit S	et al	Where are we in the world of proteomics and bioinformatics?	2012	Conference Abstract
Bisazza K	et al	Global Proteomic Analysis of Bone Biopsies Reveal Pathway Changes Over 12-Months in an Ovine Model of Osteoporosis	2024	Conference Abstract
Bostanci N	et al	Revisiting "-omics" in Oral Health and Disease	2020	Editorial
Boulos M	et al	Hidden in the Fat: Unpacking the Metabolic Tango Between Metabolic Dysfunction-Associated Steatotic Liver Disease and Metabolic Syndrome	2025	Review
Brumpton BM	et al	The HUNT study: A population-based cohort for genetic research	2022	Missing one or more keywords
Cahill LE	et al	Nutrigenomics: A possible road to personalized nutrition	2019	Book chapter
Calciolari E	et al	Proteomic and Transcriptomic Approaches for Studying Bone Regeneration in Health and Systemically Compromised Conditions	2020	Review
Chai JH	et al	Oligo/polysaccharides from Cyathula officinalis and Achyranthes bidentata: a review of structures and bioactivities	2024	Review
Chandwar K	et al	What does artificial intelligence mean in rheumatology?	2024	Review
Chen D	et al	Identification of Key Osteoporosis Genes Through Comparative Analysis of Men's and Women's Osteoblast Transcriptomes	2023	Non-clinical
Christenson SA	et al	Chronic obstructive pulmonary disease	2022	Review
Civitelli R	et al	A Most Rewarding Experience	2022	Editorial
Cui W	et al	Exploring the neuroprotective effect and potential mechanism of Shenrong Guben Huanshao Pill based on multi-omics analysis	2025	Missing one or more keywords
Curtis EM	et al	Epigenetic regulation of bone mass	2022	Review
Das A	et al	Integrative single-cell RNA-seq and ATAC-seq identifies transcriptional and epigenetic blueprint guiding osteoclastogenic trajectory	2025	No Human
Daswani B	et al	"Omics" Signatures in Peripheral Monocytes from Women with Low BMD Condition	2018	Review
Datta P	et al	An 'omics approach towards CHO cell engineering	2013	Review
de Kock L	et al	Combined transcriptome and proteome profiling of SRC kinase activity in healthy and E527K defective megakaryocytes	2021	Letter
Ding Y	et al	Integrative Omics Reveals Glutamine Catabolism-Driven Apoptotic Suppression in Monocytes upon Mechanical Unloading	2025	Missing one or more keywords
DuranFrigola M	et al	Drug repositioning beyond the low-hanging fruits	2017	Review
Eisfeldt J	et al	Multi-omics analysis of DNA and RNA identifies disruption of MINK1 in a balanced translocation carrier with congenital cataract and epilepsy	2023	Conference Abstract
Evans W	et al	Precision Medicine—Are We There Yet? A Narrative Review of Precision Medicine's Applicability in Primary Care	2024	Review
Fan W	et al	A Study of Skeletal Stem Cell Dynamics and Its Potential Applications in the Design of a Titanium Implant for Senile Osteoporosis	2025	No Human
Fan X	et al	Spatiotemporal Dynamics of Osteoarthritis: Bridging Insights from Bench to Bedside	2025	Review
Feng K	et al	Multi-omics analysis of bone marrow mesenchymal stem cell differentiation differences in osteoporosis	2023	Irrelevant

Feng S	et al	Application of Single-Cell and Spatial Omics in Musculoskeletal Disorder Research	2023	Review
Ferreira R	et al	Glycolysis: Tissue-Specific Metabolic Regulation in Physio-pathological Conditions	2023	Book chapter
Foessl I	et al	Bone Phenotyping Approaches in Human, Mice and Zebrafish – Expert Overview of the EU Cost Action GEMSTONE (“GEnomics of MusculoSkeletal traits TranslatiOnal NEtwork”)	2021	Review
Fu L	et al	Repurposing non-oncology small-molecule drugs to improve cancer therapy: Current situation and future directions	2022	Review
Gao S	et al	New technologies for bone diseases	2023	Book chapter
Gemmati D	et al	“Bridging the Gap” Everything that Could Have Been Avoided If We Had Applied Gender Medicine, Pharmacogenetics and Personalized Medicine in the Gender-Omics and Sex-Omics Era	2020	Review
Gong A	et al	Calycosin orchestrates osteogenesis of Danggui Buxue Tang in cultured osteoblasts: Evaluating the mechanism of action by omics and chemical knock-out methodologies	2018	No Human
Grassi F	et al	Report and Abstracts of the 18th Meeting of the Interuniversity Institute of Myology: Virtual meeting, October 21–24, 2021	2021	Conference Abstract
Grimm M	et al	The Lichens’ Microbiota, Still a Mystery?	2021	Review
Gruber M	et al	Effect of teriparatide treatment on amino acid metabolism during osteoblast differentiation	2025	Conference Abstract
Gu ZX	et al	Research advances in the study of traditional Chinese medicine formula granules on signaling pathway-mediated disease mechanisms	2025	Review
Guan Q	et al	Bone-Derived Factors: Regulating Brain and Treating Alzheimer’s Disease	2025	Review
Haddad S	et al	Future Data Points to Implement in Adult Spinal Deformity Assessment for Artificial Intelligence Modeling Prediction: The Importance of the Biological Dimension	2023	Missing one or more keywords
Hamamoto R	et al	Application of artificial intelligence for medical research	2021	Editorial
Ho ST	et al	Characterizing therapeutic effects of velvet antler using different omics strategies	2025	Review
Hoda U	et al	Characteristics of the frequent exacerbator in U-BIOPRED adult severe asthma cohort	2015	Conference Abstract
Holzinger ER	et al	Integrating heterogeneous high-throughput data for meta-dimensional pharmacogenomics and disease-related studies	2012	Review
Hsu Y	et al	Proteogenomic signatures of Osteoporotic Fractures and Risk Prediction: A multi-omics approach for musculoskeletal biology	2024	Conference Abstract
Huang J	et al	Multi-Omics Integrative Analyses Identified Two Endotypes of Hip Osteoarthritis	2024	No Human
Imai Y	et al	Integrative single-cell multi-omics analysis captures a role of IRF8 as a gatekeeper for osteoclastogenesis	2025	Editorial
Ji J	et al	Gut Microbiota in Primary Osteoporosis: a Systematic Review	2024	Letter
Jia M	et al	Synergistic effects of compound plant extracts and Lactobacillus plantarum on osteogenesis: prebiotic potential and mechanistic insights from metabolomics and molecular docking	2025	No Human
Jiang L	et al	Analysis of subgingival micro-organisms based on multi-omics and Treg/Th17 balance in type 2 diabetes with/without periodontitis	2022	Missing one or more keywords
Jiang T	et al	Multi-omics and bioinformatics for the investigation of therapeutic mechanism of roucongrong pill against postmenopausal osteoporosis	2025	Missing one or more keywords
Julkunen H	et al	Comprehensive interaction modeling with machine learning improves prediction of disease risk in the UK Biobank	2025	Missing one or more keywords
Karasik D	et al	A whole genome sequencing study to identify novel genetic variants associated with lean mass: Multi-ethnic meta-analysis	2021	Conference Abstract
KhoshnamRad N	et al	Transforming IBD care: the future of personalized therapy through multi-omics and pharmacogenomics	2025	Review
Kiel D	et al	The Musculoskeletal Knowledge Portal: Making Omics Data Useful to the Broader Scientific Community	2020	Review
Kim S	et al	Recent development in bioinformatics for utilizing omics data	2014	Editorial
Klingerstorff V	et al	Chenodeoxycholic acid regulates osteoblast metabolism by FXR-dependent and independent mechanisms	2023	Conference Abstract

KodriÄ® K	et al	P4Â medicine and osteoporosis: aÂ systematic review	2016	Review
Lee S	et al	Use of omics data in fracture prediction: a scoping and systematic review in horses and humans	2021	Review
Li C	et al	Jiangu granules ameliorate postmenopausal osteoporosis via rectifying bone homeostasis imbalance: A network pharmacology analysis based on multi-omics validation	2024	No Human
Li H	et al	Single-cell multi-omics identify novel regulators required for osteoclastogenesis during aging	2024	No Human
Li JP	et al	Tri-dimensional omics analysis on effect of Zhuanggu Zhitong capsule against experimental postmenopausal osteoporosis	2014	No Human
Li N	et al	Plasma proteome profiling combined with clinical and genetic features reveals the pathophysiological characteristics of β-thalassemia	2022	Missing one or more keywords
Li Q	et al	Integrated metagenomic and metabolomic analyses of the effects of total flavonoids of Rhizoma Drynariae on reducing ovariectomized-induced osteoporosis by regulating gut microbiota and related metabolites	2025	Irrelevant
Li Q	et al	From Genomics to Metabolomics: Molecular Insights into Osteoporosis for Enhanced Diagnostic and Therapeutic Approaches	2024	Review
Li S	et al	Multi-omics joint analysis reveals the mechanism underlying Chinese herbal Yougui Pill in the treatment of knee osteoarthritis	2025	Irrelevant
Li X	et al	Current cutting-edge omics techniques on musculoskeletal tissues and diseases	2025	Review
Li X	et al	Integrative multi-omics and network pharmacology reveal angiogenesis promotion by Quan-Du-Zhong Capsule via VEGFA/PI3K-Akt pathway	2025	Missing one or more keywords
Li YR	et al	Retrospective study on intervention of traditional Chinese medicine in osteoporosis and related pain diseases	2025	Missing one or more keywords
Lin D	et al	Integrative analysis of multiple diverse omics datasets by sparse group multitask regression	2014	No Human
Lin L	et al	Systems biology of meridians, acupoints, and Chinese Herbs in disease,	2012	Missing one or more keywords
Lin X	et al	Using multi-omics to explore the role of gut microbiota in pathogenesis of postmenopausal bone health	2019	Conference Abstract
Ling Z	et al	Co-morbid mechanisms of intervertebral disc degeneration and osteoporosis: biomechanical coupling and molecular pathways synergistically driving degenerative lesions	2025	Review
Liu D	et al	Repurposing Acebutolol for Osteoporosis Treatment: Insights From Multi-Omics and Multi-Modal Data Analysis	2025	Irrelevant
Liu D	et al	Multi-omics analyses of drug repurposing reveal Acebutolol and Amiloride for osteoporosis treatment	2022	Irrelevant
Liu M	et al	Molecular endotypes in musculoskeletal disorders: a systematic review	2025	Conference Abstract
Long J	et al	Advances in untargeted metabolomics research on osteoporosis	2025	Review
Lotz M	et al	Value of biomarkers in osteoarthritis: Current status and perspectives	2014	No Human
Lotz M	et al	Republished: Value of biomarkers in osteoarthritis: current status and perspectives	2014	Missing one or more keywords
Lv H	et al	Metabolomics and its application in the development of discovering biomarkers for osteoporosis research	2016	Review
Mahan B	et al	Isotope metallomics approaches for medical research	2020	Review
Malavašič P	et al	Recent Advances in Experimental Functional Characterization of GWAS Candidate Genes in Osteoporosis	2025	Review
Marc J	et al	System medicine and personalised laboratory medicine in bone diseases	2016	Conference Abstract
Mathioudakis AG	et al	COPD phenotypes and biomarkers: Introducing personalised medicine	2013	Missing one or more keywords
Min Y	et al	Cohort Profile: WELL Living Laboratory in China (WELL-China)	2021	Note
Moayyeri A	et al	Cohort profile: Twinsuk and healthy ageing twin study	2013	Missing one or more keywords
Mobasheri A	et al	Bone and cartilage talk together	2022	Conference Abstract

Murphy C	et al	Emerging role of extracellular vesicles in musculoskeletal diseases	2018	Review
Nava-Gonzalez E	et al	Mini-review: The contribution of intermediate phenotypes to gxe effects on disorders of body composition in the new OMICS era	2017	Review
Ndlovu T	et al	French and Mediterranean-style diets: Contradictions, misconceptions and scientific facts-A review	2019	Review
Nusrat S	et al	Epigenetic Dysregulation and Osteocyte Senescence: Convergent Drivers of Osteosarcopenia in Aging Bone and Muscle	2025	Review
Panahi N	et al	Metabolomic biomarkers of low BMD: a systematic review	2021	Review
Park SY	et al	Evolving Concept of Severe Asthma: Transition from Diagnosis to Treatable Traits	2022	Missing one or more keywords
Pichard L	et al	Establishment of a collection of human pluripotent stem cell lines (iPSC) from mesenchymal stem cells (MSC) from three healthy elderly donors	2021	Missing one or more keywords
Prasad B	et al	S8.4 - Integrated quantitative proteomics and metabolomics approach for discovery and validation of UGT2B17 biomarker to predict drug metabolism	2020	Conference Abstract
Qiao X	et al	Gut microbial community and fecal metabolomic signatures in different types of osteoporosis animal models	2024	No Human
Rauner M	et al	Perspective of the GEMSTONE Consortium on Current and Future Approaches to Functional Validation for Skeletal Genetic Disease Using Cellular, Molecular and Animal-Modeling Techniques	2021	Review
Reisz JA	et al	When nature’s robots go rogue: exploring protein homeostasis dysfunction and the implications for understanding human aging disease pathologies	2018	Review
Reppe S	et al	Omics analysis of human bone to identify genes and molecular networks regulating skeletal remodeling in health and disease	2017	Review
Salmi A	et al	An integrative bioinformatics approach to decipher adipocyte-induced transdifferentiation of osteoblast	2022	Non-clinical
Salvioli S	et al	Biomarkers of aging in frailty and age-associated disorders: State of the art and future perspective	2023	Review
Santoro A	et al	Nutrition as a tool to counteract inflammaging: results and challenges from the nu-age project	2022	Conference Abstract
Santos L	et al	Multiomic analysis of stretched osteocytes reveals processes and signalling linked to bone regeneration and cancer	2021	Irrelevant
Schmidt J	et al	Integrative tissue and plasma proteomics revealed liquid biopsy-accessible indicative biomarkers for impaired bone fracture healing in type 2 diabetes mellitus	2023	Conference Abstract
Schurman CA	et al	Molecular and Cellular Crosstalk between Bone and Brain: Accessing Bidirectional Neural and Musculoskeletal Signaling during Aging and Disease	2023	Missing one or more keywords
Selvamuthukumaran M	et al	Novel approaches in food functions based on nutrigenomics research	2017	Book chapter
Semeraro M	et al	High concentrations of primary bile acids alter osteoblast metabolism and reduce bone mineralisation	2023	Conference Abstract
Setoyama D	et al	Comparative Analysis of Primary Sarcopenia and End-Stage Renal Disease–Related Muscle Wasting Using Multi-Omics Approaches	2025	Irrelevant
Shang G	et al	Multi-omics analysis of kidney, bone and bone marrow explored potential mechanisms of Erzhi Wan against osteoporosis with kidney-Yin deficiency	2024	Irrelevant
Shen CL	et al	Tocotrienol Supplementation Led to Higher Serum Levels of Lysophospholipids but Lower Acylcarnitines in Postmenopausal Women: A Randomized Double-Blinded Placebo-Controlled Clinical Trial	2021	Missing one or more keywords
Shen J	et al	Mitochondrial Transplantation: A Paradigm Shift in Osteoporosis Therapy	2025	Review
Shida T	t al	Identification of metabolites associated with the development of sarcopenia in older women: A longitudinal nested case–control study	2025	Missing one or more keywords
Snodgrass J	et al	Minimally invasive biomarkers in human population biology research	2022	Missing one or more keywords
Spira A	et al	Precancer atlas to drive precision prevention trials	2017	Review
Sreejalekshmi KG	et al	Space Biosciences: Translational Research for Space, Benefitting Life on Earth	2024	Book chapter

Strathmann E	et al	Epigenetic regulation of plastin 3 expression by the macrosatellite DXZ4 and the transcriptional regulator CHD4	2023	Missing one or more keywords
Tan L	et al	Identification of novel genes and regulatory network modules for bone mineral density by integrated analyses of transcriptome	2013	Conference Abstract
TarÃn J	et al	Infertility etiologies are genetically and clinically linked with other diseases in single meta-diseases	2015	Editorial
Teixeira Rosa JT	et al	Fish Models of Induced Osteoporosis	2021	Review
Teng Z	et al	Deciphering the chromatin spatial organization landscapes during BMMSC differentiation	2023	Missing one or more keywords
To K	et al	Exploring the molecular mechanism of type XV osteogenesis imperfecta caused by WNT1 mutation	2024	Conference Abstract
Tobias JH	et al	Opportunities and Challenges in Functional Genomics Research in Osteoporosis: Report From a Workshop Held by the Causes Working Group of the Osteoporosis and Bone Research Academy of the Royal Osteoporosis Society on October 5th 2020	2020	Conference Abstract
Tobias JH	et al	Editorial: Recent Advances in the Genetics of Osteoporosis	2021	Editorial
Venkataraman A	et al	Decoding senescence of aging single cells at the nexus of biomaterials, microfluidics, and spatial omics	2024	Review
Vineis P	et al	The biology of inequalities in health: The LIFEPATH project	2017	Missing one or more keywords
Wang J	et al	Emerging applications of feature selection in osteoporosis research: from biomarker discovery to clinical decision support	2025	Review
Wang JS	et al	SP7: from Bone Development to Skeletal Disease	2023	Review
Wang JS	et al	Pathways Controlling Formation and Maintenance of the Osteocyte Dendrite Network	2022	Review
Wang JS	et al	SP7: from Bone Development to Skeletal Disease	2023	Conference Abstract
Wang W	et al	Kunxinning granules alleviate perimenopausal syndrome by supplementing estrogen deficiency	2025	Missing one or more keywords
Wang X	et al	An updated overview of the search for biomarkers of osteoporosis based on human proteomics	2024	Review
Wang Y	et al	Qing'e Pill rectifies bone homeostasis imbalance in diabetic osteoporosis via the AGE/RAGE pathway: A network pharmacology analysis and multi-omics validation	2025	No Human
Wei Q	et al	Function-oriented mechanism discovery of coumarins from Psoralea corylifolia L. in the treatment of ovariectomy-induced osteoporosis based on multi-omics analysis	2024	No Human
Weigelt J	et al	Structural genomics-Impact on biomedicine and drug discovery	2010	Review
Wen Y	et al	Mechanisms of Anemarrhenae Rhizoma in Treating Osteoporosis in Rats: An Integrated Metabolomics and Transcriptomics Analysis	2025	No Human
Westendorf JJ	et al	The Musculoskeletal Knowledge Portal: improving access to multi-omics data	2022	Note
Wu J	et al	FAM210A Maintains Cardiac Mitochondrial Homeostasis Through Regulating Letm1-dependent Ca(2+) Efflux	2021	Conference Abstract
Wu P	et al	Cohort Profile: Taiwan Kidney Outcome (TAKO) and Taiwan Kidney Outcome Omics (TAKOO) cohorts	2025	Missing one or more keywords
Wu Q	et al	Bridging Genomic Research Disparities in Osteoporosis GWAS: Insights for Diverse Populations	2025	Review
Wu Y	et al	Osteocytes: master orchestrators of skeletal homeostasis, remodeling, and osteoporosis pathogenesis	2025	Review
Wu Y	et al	Insight into the physiopathologic mechanism for the coexistence of depression and osteoporosis	2015	Missing one or more keywords
Xie X	et al	The gut microbiota in osteoporosis: dual roles and therapeutic prospects	2025	Review
Xu T	et al	Editorial: The role of probiotics, postbiotics, and microbial metabolites in preventing and treating chronic diseases	2023	Editorial
Xu Y	et al	Recent advances in the epigenetics of bone metabolism	2021	Review
Xu Z	et al	Mechanisms of estrogen deficiency-induced osteoporosis based on transcriptome and DNA methylation	2022	No Human
Xue S	et al	Integrated fecal macrogenomic and metabolomic analyses reveal celiac disease flora and metabolic profiles associated with Chinese populations	2025	Missing one or more keywords

Yang J	et al	Discovery of potential biomarkers for osteoporosis diagnosis by individual omics and multi-omics technologies	2023	Review
Yang J	et al	Multi-omics Mendelian Randomization and Single-Cell Analysis Identify Novel Therapeutic Targets for Osteosarcopenia	2025	No Open Access
Yang M	et al	Immunotherapies for Aging and Age-Related Diseases: Advances, Pitfalls, and Prospects	2025	Review
Yang T	et al	A road map for understanding molecular and genetic determinants of osteoporosis	2020	Review
Yang Y	et al	Multi-omics knowledge spectrum of osteoporosis: A bibliometric and visual analysis	2025	Review
Yin Y	et al	A Multi-Omics Analysis Reveals Anti-Osteoporosis Mechanism of Four Components from Crude and Salt-Processed Achyranthes bidentata Blume in Ovariectomized Rats	2022	No Human
You Y	et al	Interactions between the gut microbiota and immune cell dynamics: novel insights into the gut-bone axis	2025	Review
Yu H	et al	Impact of monotherapy and combination therapy with glucagon-like peptide-1 receptor agonists on exosomal and non-exosomal MicroRNA signatures in type 2 diabetes mellitus: a systematic review	2025	Review
Yuan S	et al	Epidemiology of sarcopenia: Prevalence, risk factors, and consequences	2023	Review
Yuan Y	et al	Intelligent biomaterials for chronic diseases caused by aging	2024	Review
Zargar S	et al	Silicon: A multitalented micronutrient in OMICS perspective - an update	2012	Review
Zhang J	et al	Iron-lead mixed exposure causes bone damage in mice: A multi-omics analysis	2025	No Human
Zhang JG	et al	Integrative analysis of transcriptomic and epigenomic data to reveal regulation patterns for BMD variation	2015	Missing one or more keywords
Zhang L	et al	Developmental allometry of articular chondrocytes from hypertensive rats: a possible link with dysregulated IGF signaling	2025	Conference Abstract
Zhang L	et al	Identification of New Biomarkers and Potential Therapeutic Targets in Clinical Osteoporosis Using Omics Technologies	2024	No Open Access
Zhang X	et al	IMPC-based screening revealed that ROBO1 can regulate osteoporosis by inhibiting osteogenic differentiation	2024	Missing one or more keywords
Zhang X	et al	Sequence variants associated with variation in lean mass: Multi-ethnic metaanalysis	2022	Conference Abstract
Zhao Y	et al	Beyond Bone Loss: A Biology Perspective on Osteoporosis Pathogenesis, Multi-Omics Approaches, and Interconnected Mechanisms	2025	Review
Zhao Z	et al	Enhancing Akkermansia growth via phytohormones: a strategy to modulate the gut-bone axis in postmenopausal osteoporosis therapy	2025	Missing one or more keywords
ZrimÅ¡ek M	et al	Integrated functional-omics framework of genome-wide association study (GWAS) of osteoporosis traits postulate FUBP3 and ETS2 as critical factors of bone metabolism	2017	Conference Abstract
		4th International Workshop on Predictive Intelligence in Medicine (PRIME 2021) held in conjunction with MICCAI 2021	2021	Conference Abstract
		Real-World Long-Term Musculoskeletal and Extra-Skeletal Outcomes of Obesity	2025	No Open Access
		Osteocytes: master orchestrators of skeletal homeostasis	2025	Review
		Key genes linking gut microbiota	2025	Missing one or more keywords
		Lipid Metabolism	2024	Missing one or more keywords
		From Exposome to Health Outcomes: A Population-Based Approach to Understanding and Mitigating Early Menopause in Singapore's Diverse Communities	2025	Missing one or more keywords
		Study of the Key Techniques of Prevention and Treatment of Osteoporotic Refracture	2023	Missing one or more keywords
		Guangzhou Nutrition and Health Study (GNHS): A Multiomics-based Study	2017	Missing one or more keywords
		SSIEM 2016 Annual Symposium - Abstracts	2016	Conference Abstract

The exclusion criteria were defined to ensure the relevance, accessibility, and methodological quality of the included studies. Specifically, the following were excluded: i) articles missing one or more keywords, as they were not adequately indexed and potentially not aligned with the search strategy; ii) book chapters, editorials, letters, or notes, as they do not represent original research contributions subjected to full peer review; iii) conference abstracts or proceedings, which are often characterized by limited information and preliminary data; iv) studies not relevant to the main topic or lacking translational relevance, as they were not directly applicable to the study objectives; v) studies not conducted on human subjects, in order to maintain a focus on clinical relevance; vi) articles not available in open access, to ensure full accessibility and data verifiability; vii) narrative reviews, systematic reviews, or meta-analyses, since the analysis was exclusively focused on original research studies.
